# Occupational exposure to whole-body vibrations and pregnancy complications: a nationwide cohort study in Sweden

**DOI:** 10.1136/oemed-2020-106519

**Published:** 2020-06-03

**Authors:** Helena Skröder, Hans Pettersson, Maria Albin, Per Gustavsson, Lars Rylander, Filip Norlén, Jenny Selander

**Affiliations:** 1 Institute of Environmental Medicine, Karolinska Institutet, Stockholm, Sweden; 2 Department of Public Health and Clinical Medicine, Umeå University, Umeå, Sweden; 3 Division of Occupational and Environmental Medicine, Lund University, Lund, Sweden

**Keywords:** hygiene / occupational hygiene, epidemiology, female reproductive effects and adverse pregnancy outcomes, vibration

## Abstract

**Objectives:**

Pregnancy complications are common contributors to perinatal mortality and morbidity. Still, the cause(s) of gestational hypertensive disorders and diabetes are largely unknown. Some occupational exposures have been inconsistently associated with pregnancy complications, but exposure to whole-body vibrations (WBV) has been largely overlooked even though it has been associated with adverse birth outcomes. Therefore, the aim was to assess whether occupational WBV exposure during pregnancy is associated with pregnancy complications in a nationwide, prospective cohort study.

**Methods:**

The Fetal Air Pollution Exposure cohort was formed by merging multiple Swedish, national registers containing information on occupation during pregnancy and diagnosis codes, and includes all working women who gave birth between 1994 and 2014 (n=1 091 044). WBV exposure was derived from a job-exposure matrix and was divided into categories (0, 0.1–0.2, 0.3–0.4 and ≥0.5 m/s^2^). ORs with 95% CIs were calculated using logistic regression adjusted for potential confounders.

**Results:**

Among women working full time (n=646 490), we found increased risks of all pregnancy complications in the highest exposure group (≥0.5 m/s^2^), compared with the lowest. The adjusted ORs were 1.76 (95% CI 1.41 to 2.20), 1.55 (95% CI 1.26 to 1.91) and 1.62 (95% CI 1.07 to 2.46) for preeclampsia, gestational hypertension and gestational diabetes, respectively, and were similar in all sensitivity analyses. There were no clear associations for part-time workers.

**Conclusions:**

The results suggest that women should not be exposed to WBV at/above the action limit value of 0.5 m/s^2^ (European directive) continuously through pregnancy. However, these results need further confirmation.

**Key messages**

**What is already known about this subject?**

- An increasing number of women are choosing to work in industries where whole-body vibration exposure is common, but still, very little is known about risks of such exposure during pregnancy.

**What are the new findings?**

- This is the first prospective study to address this issue, and is also unique in terms of the detailed exposure assessment, sample size and in ability to adjust for multiple, quantitatively assessed, occupational exposures.
- Exposed women (mainly truck drivers, forklift and heavy machine operators) working full time had an increased risk of both preeclampsia, gestational hypertension and gestational diabetes.
- The results are alarming, as the associations were apparent below the current exposure limit value set by the European Union.

**How might this impact on policy or clinical practice in the foreseeable future?**

- The results are likely to lay the foundation of future risk assessment of pregnant women exposed to whole-body vibrations.
- If confirmed by others, the results indicate that women should not be continuously exposed to whole-body vibrations throughout pregnancy, which supports development of policies for reassignment or pregnancy allowance for at least part of the pregnancy.

## Introduction

Pregnancy complications like preeclampsia, gestational hypertension and gestational diabetes are common causes of perinatal mortality and morbidity as they increase the risk of both preterm birth and fetal growth restriction.^1–3^ Preeclampsia and gestational hypertension complicate between 0.2%–9.2% and 1.8%–4.4% of all pregnancies worldwide, respectively, and the incidence varies widely between countries (higher in North/South America and lower in Africa).^4^ Both disorders are characterised by new onset (after 20 weeks of gestation) hypertension, but preeclampsia is further accompanied by one or more of the following conditions: proteinuria, dysfunction of other maternal organ (either liver, kidney or neurological), haematological involvement and/or uteroplacental dysfunction.^5^ Gestational diabetes is characterised by insulin resistance and affects around 5.8%–12.9% of all pregnancies.^6^ Again, the variation in incidence both within and between countries is wide (highest in Middle East/North Africa and lowest in Europe), partly due to population characteristics, but also because of differences in screening and diagnostic criteria.

Risk factors for these pregnancy complications include having had such a complication in a previous pregnancy, overweight, high maternal age, nulliparity, type 1 and type 2 diabetes, as well as multiple births.^2^ Still, prediction models for pregnancy complications vary widely in performance,^7 8^ indicating that there may be additional, unknown factors that influence the risk. Some occupational exposures, such as noise and physically strenuous work, have been associated with preeclampsia, but results are conflicting and too limited to draw any conclusions from.^9 10^

An additional occupational factor, that has not been well studied in this matter but is still advised against (during pregnancy) by the Swedish Work Environment Authority,^11^ is exposure to whole-body vibrations (WBV). Exposure is common among vehicle and heavy machine operators in industry, construction, agriculture, forestry and transportation^12 13^ and involves about 1%–2% of all working women in Sweden^14–16^ and the UK.^17^ WBV exposure has previously been associated with adverse birth outcomes in a few epidemiological studies^18–20^ and changes in uterine blood flow and hormone levels in an animal experimental study.^21^ Yet, there is only one small study (case control, n=102 cases of preeclampsia and n=99 cases of gestational hypertension) assessing the risk of hypertensive disorders during pregnancy in relation to WBV exposure, which showed inconclusive results.^9^ However, there are no such studies concerning gestational diabetes. There is therefore a strong need for more epidemiological studies investigating this, to clarify whether there is an increased risk of pregnancy complications in relation to WBV exposure during pregnancy.

Therefore, the aim of the present study was to investigate whether occupational exposure to WBV during pregnancy is associated with the risk of preeclampsia, gestational hypertension and/or gestational diabetes in a nationwide cohort study of Swedish women.

## Subjects and methods

### Study population

The Fetal Air Pollution Exposure cohort was formed by merging multiple Swedish national registers, and contains information regarding all registered births between 1994 and 2014 (n=2 113 336).^22^ The Medical Birth Register has a 98%–99% coverage and contains data regarding, for example, maternal age, height, weight, occupation, smoking habits, parity and country of birth, which is collected at the first visit to the prenatal care clinics (usually around gestational week 10).^23^ The register also has information on diagnoses during pregnancy. The Longitudinal Integrated Database for Health Insurance and Labor Market Studies (LISA) has full coverage for all residents above 16 years of age and contains data on education (highest degree), which may be used as proxy for socioeconomic status.

For 1 796 078 pregnancies, an occupation was stated at the visit to the prenatal care clinic and could thereby be included for exposure assessment. However, 705 034 pregnancies were excluded due to: the woman being a student or unemployed at the time of pregnancy (n=319 401), stating ‘not working’ (n=109 153) or missing employment information (n=88 021) even though an occupation was reported, providing a titles/code that is too general to code (such as ‘consultant’) or unique (n=151 378; not included due to limited coding resources), or being part of flight crew (required to be reassigned to ground work to avoid radiation over 1 ms,^11^ leading to exposure misclassification; n=2685). In addition, we excluded multiple births (n=34 396), leaving a final sample of 1 091 044 pregnancies.

### Exposure assessment

The mother’s occupation was entered as free text at the prenatal care clinic interview (around gestational week 10), and this was coded by the research group into occupational codes according to the occupational classifications of the National Labour Market Board (Arbetsmarknadsstyrelsens yrkesklassificering), based on the International Standard Classification of Occupations, ISCO-88-code system, as described elsewhere.^24^ Exposure to WBV was assessed quantitatively through a job-exposure matrix (JEM), developed for this study. For each occupational code, the daily 8-hour energy-equivalent frequency-weighted exposure value (m/s^2^) was estimated by measured vibration levels and duration of exposure, according to the international standard (ISO 2631-1). The daily vibration levels were based on a large number of measurements from different vehicles and heavy equipment machinery collected from scientific articles (n=32), occupational medicine clinic reports (n=19), reports from the former National institute for Workers Life in Sweden and the national Swedish vibration database and literature reviews (n=3). The classification was performed by one occupational hygienist and one researcher specialising in vibration exposure. The JEM was constructed for 5-year periods (1994–1998, 1999–2003, 2004–2008, 2009–2013, 2014–2016), allowing the WBV exposure to vary over time within each occupational code. There were 31 occupations identified with exposure ≥0.1 m/s^2^, and from all the measurement data, 78 daily 8-hour energy-equivalent frequency-weighted values were calculated for at least one of the time-periods for each occupation with WBV exposure.

In addition to WBV, exposure to mechanical shocks was also included in the JEM. However, this was only assessed as yes/no, in contrast to the more detailed WBV exposure (m/s^2^). The occupational hygienist and researcher performing the exposure assessment were blinded to the outcomes, since the exposure assessment of each occupation was made before the JEM was attached to the cohort. Exposure to WBV and mechanical shocks was merged with the Medical Birth Register data through the occupational code according to the year of pregnancy. Exposure data were available for all included women with coded occupations that were specific enough for vibration assessment (n=1 091 044).

### Outcome definitions

Information on pregnancy complications, including preeclampsia, gestational hypertension and gestational diabetes, was collected from the Medical Birth Register. The diagnoses used for outcome classification were coded based on the Swedish versions of the International Classification of Diseases (ICD; version 9 until 1996 and version 10 thereafter), and were entered by the physician in charge at discharge from the hospital.

Preeclampsia was defined as ICD-9 codes 642E, 624F and 642H, and ICD-10 codes O11.9 and O14.0-O14.9. The clinical definition of preeclampsia during the time of the study included systolic blood pressure ≥140 and/or diastolic blood pressure ≥90 (new onset, after gestational week 20), together with proteinuria (≥0.3 g per 24 hours). We also included women with eclampsia (ICD-9 code 642G and ICD-10 codes O15.0–O15.9) in this outcome, as this must be preceded by preeclampsia.

Gestational hypertension was defined as blood pressure ≥140/90 (also new onset), and was coded as ICD-9 codes 642, 642D and ICD-10 code O13.9. In addition, all women diagnosed with preeclampsia were included in this outcome since hypertension is one of the criteria for setting a preeclampsia diagnosis.

Gestational diabetes was identified though ICD-9 code 648A, 648W and ICD-10 codes O24.4 and O24.9. The criteria for this diagnosis have varied both over time and between counties within Sweden, and is either based on fasting blood glucose concentration or on blood glucose concentration after an oral glucose tolerance test.

### Covariates

In the Medical Birth Register, there is information about maternal characteristics collected during the visits to the prenatal care clinics (weight (kg), height (cm), age (years), parity (birth order of the child), nationality (country or continent), family structure (living with father, single mother, or other), and smoking habits (non-smoking, 1–9 cigarettes/day,≥10 cigarettes/day)). We calculated maternal body mass index (BMI; kg/m^2^) and categorised this into underweight (<18.5 kg/m^2^), normal weight (18.5–24.9 kg/m^2^), overweight (25–29.9 kg/m^2^) and obese (≥30 kg/m^2^),^25^ and also classified nationality into four groups (Swedish, European, Other, Unknown/missing). The LISA register provided information on maternal educational level (seven levels that were further categorised into four groups based on the highest degree:≤elementary, high-school, higher education <3 years, higher education ≥3 years) as well as marital status (married/registered partner, divorced, not married or widowed) .

Regarding additional occupational exposures that may correlate with exposure to WBV, we assessed potential confounding from exposure to noise, combustion particles, job strain and physically strenuous work. These exposures were all assessed through different JEM:s^22 26 27^ and matched by occupational codes as mentioned above for WBV. The exposure to noise was based on measurement reports collected from occupational medicine clinics, occupational health services and large companies, and was divided into five categories: <70, 70–74, 75–79, 80–84 or ≥85 dB. Exposure to combustion particles was also based on measurements from different work places, and was in this study expressed as yes/no based on exposure to asphalt, diesel, polycyclic aromatic hydrocarbons, lead and/or other combustion particles.^22^ For job strain and physically strenuous work, an index with scores from 1 to 6 and 1–10, respectively, was calculated based on answers from the work environment survey performed by the Swedish Work Environment Authority every other year.^14^ The survey is sent to a sample of the working population (ages 16–74, n≈4000–12 000) and includes questions regarding control and demands (job strain), as well as turning, lifting, bending, heavy breathing, working with hands above the head or with heavy objects, and repeated movements (physically strenuous work). The average of the indices for physical exertion mentioned above was calculated and used as exposure to physically strenuous work. For job strain, the averages of the indices for demands and control were calculated, split at the median and combined into high/low groups (four groups: low demands and high control (low strain), high demands and low control (high strain), low demands and low control (passive), and high demands and high control (active)), in accordance with the Karasek job strain model.^28^

### Statistical methods

All statistical analyses were performed in Stata/SE (V.14.2; StataCorp, Texas, USA). For estimating the risk of pregnancy complications in relation to WBV exposure, we calculated ORs (crude and adjusted (aOR)) with 95% CIs, using logistic regression and complete subject analyses. Exposure to WBV was categorised into four groups (0 (no exposure), 0.1–0.2 (low exposure), 0.3–0.4 (medium exposure) and ≥0.5 m/s^2^ (moderately high exposure)), and mechanical shocks assessed as yes/no. We used the variance inflation factor (VIF) to assess if the variance of the estimates is increased due to collinearity, which is calculated by regressing all covariates against each other and plugging the R^2^ values into VIF=1/(1−R_i_^2^). This was <4 for all included variables, which is usually considered low (no evidence of variance inflation).

Variables that were associated with both exposure and outcome (any of preeclampsia, gestational hypertension and/or gestational diabetes; p<0.05) were kept for further assessment, and these included maternal BMI, age, living situation, marital status, parity, educational level, smoking habits, nationality, calendar year and exposure to noise, combustion particles, physically strenuous work and job strain. In the next step, we added the variables one by one (in order of significance for the association with WBV) to logistic models for all outcomes (preeclampsia, gestational hypertension and gestational diabetes). Variables were kept if they changed the estimate for WBV exposure (any category) by >5% in any of the models, leaving maternal BMI, age, parity, educational level, smoking habits, nationality, calendar year, and exposure to noise, combustion particles, physically strenuous work and job strain.

For analyses with gestational hypertension as the outcome, we excluded women with prior hypertension diagnosis (ICD-9 401–405, 642C, 642H and 642X, and ICD-10 O10–O11, O16 and I10–I15). Similarly, we excluded women with diabetes before pregnancy (ICD-9 250, 790C, E10–E14, 648A, and ICD-10 O24.0–24.3) from the analyses with gestational diabetes as the outcome. These diagnoses were also entered by the responsible physician at discharge from the hospital and are therefore included in the Medical Birth Register.

We stratified the analyses on full-time/part-time work to decrease the risk of exposure misclassification, since the JEM is constructed to estimate exposure for full-time workers (8 hour averages).

In sensitivity analyses, we first restricted the full-time working sample to women without higher education (≤high-school) to assure that there was no residual confounding from differences in education between exposed and unexposed women. In an additional sensitivity analysis, we included only full-time working first-time mothers, since a previous pregnancy complication increases the risk also in the next pregnancy and might affect the presence at work. In addition, we performed analyses including full-time working women who were unexposed to mechanical shocks, in order to assess the impact of WBV only. Unfortunately, we could not do the opposite since all women exposed to mechanical shocks were also exposed to WBV. Finally, we also reran the full-time model but replaced the reference group with the lowest exposed group (0.1–0.2 m/s^2^) to make sure that any association was not due to differences in other working conditions, like sitting a large part of the working day, between the reference group and the exposed groups.

## Results

In total, 1 091 044 pregnancies between 1994 and 2014 were included in the present study. Characteristics of the women are shown in table 1. On average, the women were 30 years old, 34% were overweigh (BMI ≥25 kg/m^2^), 92% were non-smokers and 65% were working full time. The frequency of preeclampsia, gestational hypertension and gestational diabetes among the included women was 3.1%, 4.1% and 0.9%, respectively. None of the included occupations had WBV exposure above the exposure limit value (1.15 m/s^2^), and only ~0.2% (n=2012) were exposed at/above the European action limit value (0.5 m/s^2^; mainly truck drivers and forklift operators). The 10 most common occupations in each exposure group are presented in table 2. Some occupations occur in two different exposure groups, as the WBV exposure has changed over time. Exposure between 0.3 m/s^2^ and 0.4 m/s^2^ was mainly found among workers in transportation (mostly drivers). Around 0.2% of the women (n=1762) were exposed to mechanical shocks, and these were also exposed to WBV (12% with WBV exposure 0.1–0.2, 32% with WBV exposure 0.3–0.4 and 56% with WBV exposure ≥0.5 m/s^2^). The characteristics are also presented separately for full-time workers in online supplementary table S1.

10.1136/oemed-2020-106519.supp1 Supplementary data

**Table 1:** Characteristics (mean±SD or %) of mothers who gave birth to singletons in Sweden during 1994–2014 and who worked full or part time, by exposure to whole-body vibrations and mechanical shocks (n=1 091 044 births)

Maternal characteristics	Whole-body vibration exposure (m/s^2^)				Exposure to mechanical shocks	
	0	0.1–0.2	0.3–0.4	≥0.5	No	Yes
	n=1 070 816 (98.2 %)	n=15 241 (1.4 %)	n=2975 (0.3 %)	n=2012 (0.2 %)	n=1 089 282 (99.8%)	n=1762 (0.2%)
Age (years) *0% missing*	30.4±4.8	30.5±4.8	30.5±4.6	28.2±4.7	30.4±4.8	29.9±4.7
BMI (%) *7% missing*						
<18.5 kg/m^2^	2.1	1.5	1.5	2.0	2.1	1.5
18.5–24.9 kg/m^2^	64.0	62.1	54.7	52.0	63.9	57.0
25–29.9 kg/m^2^	24.0	26.7	28.2	27.5	24.1	28.4
≥30 kg/m^2^	9.9	9.7	15.6	18.5	9.9	13.2
Parity (birth order of child) *0% missing*	1.8±0.90	1.7±0.91	1.8±0.95	1.7±0.91	1.8±0.90	1.7±0.83
Educational level (%) *<1% missing*						
≤Elementary	6.7	7.6	7.8	14.0	6.7	10.9
High school	47.2	48.8	61.6	79.2	47.3	55.0
Higher education <3 years	13.7	29.3	19.8	4.6	13.9	18.6
Higher education ≥3 years	32.4	14.3	10.8	2.2	32.1	15.5
Smoking (%) *1% missing*						
Non-smoking	92.0	90.9	91.8	82.7	92.0	88.2
1–9 cigarettes/day	5.9	5.9	5.3	10.6	5.9	7.4
≥10 cigarettes/day	2.1	3.2	2.9	6.7	2.1	4.5
Nationality (%) *0% missing*						
Swedish	94.1	96.1	96.9	96.6	94.1	96.5
European	3.6	2.6	2.4	2.3	3.6	2.4
Other	1.4	0.6	0.0	0.3	1.4	0.1
Unknown	0.9	0.7	0.7	0.8	0.8	1.0
Noise (%) *<1% missing*						
<70 dB	63.8	6.0	0.0	0.0	62.8	0.0
70–74 dB	18.1	91.3	40.6	0.0	19.2	9.6
75–79 dB	5.2	2.0	58.4	88.5	5.3	72.9
80–84 dB	12.0	0.7	0.0	11.5	11.8	17.5
≥85 dB	0.9	0.0	1.0	0.0	0.9	0.0
Mechanical shocks (% Yes/No) *0% missing*	0/100	1.3/98.7	19.3/80.7	48.8/51.2	0/100	100/0
Combustion particles* (% Yes/No) *<1% missing*	3.5/96.5	47.4/52.6	65.1/34.9	99.2/0.8	4.4/95.6	79.3/20.7
Job strain (%) *0% missing*						
Low strain (low demands+high control)	13.6	0.1	18.4	0.0	13.3	29.7
Active (high demands+high control)	37.4	38.8	0.0	0.0	37.3	8.6
High strain (high demands+low control)	14.2	12.0	50.1	51.2	14.3	3.3
Passive (low demands+low control)	34.8	49.1	31.5	47.8	35.1	58.4
Physically strenuous work† (index 1–6) *0% missing*	2.1±0.7	2.5±0.8	2.7±0.7	3.0±0.3	2.1±0.70	2.7±0.7

*Based on exposure to asphalt, diesel, polycyclic aromatic hydrocarbons, lead and/or other combustion particles.

†Average of indices (physically strenuous work) or combination of low/high demands/control (job strain), based on questions about control, demands, turning, lifting, bending, heavy breathing, working with hands above the head or with heavy objects, and repeated movements.

BMI, body mass index.

**Table 2:** Top 10 most common occupations (and their fraction of the exposure group) for the included women, by exposure to whole-body vibrations* and mechanical shocks

0 m/s^2^ n=1 070 816 (98.2 %)	0.1–0.2 m/s^2^ n=15 241 (1.4 %)	0.3–0.4 m/s^2^ n=2975 (0.3 %)	≥0.5 m/s^2^ n=2012 (0.2 %)	Mechanical shocks n=1762 (0.2%)
Nursing assistants (8%)	Police officers (29%)	Bus drivers (34%)	Truck drivers (51%)	Forklift operators (53%)
Teachers (6%)	Mail carriers (21%)	Mail carriers (28%)	Forklift operators (47%)	Officers (27%)
Midwives (5%)	Security guards (12%)	Officers (15%)	Heavy machine drivers (2%)	Boat machinists (6%)
Retailers (5%)	Veterinarians (9%)	Train drivers (7%)		Boat or bus attendants (3%)
Care assistants (4%)	Car drivers (8%)	Tram drivers (5%)		Ship officers (3%)
Daycare teachers (4%)	Train personnel (5%)	Fire fighters (4%)		Coast guard (3%)
Daycare workers (4%)	Newspaper distributors (5%)	Boat or bus attendants (2%)		Deck hands (3%)
Office workers (3%)	Taxi drivers (4%)	Coast guard (2%)		Heavy machine operators (3%)
Economy assistants (3%)	Parking attendants (2%)	Mineworkers (1%)		Fishers (<1%)
Cleaning staff (3%)	Train conductor (1%)	Metro drivers (<1%)		

*Occupations can be present in several categories due to changes in exposure over time.

We found that full-time working women exposed to WBV at levels≥0.5 m/s^2^ were more likely to be diagnosed with both preeclampsia (aOR=1.76, 95% CI 1.41 to 2.20), gestational hypertension (aOR=1.55, 95% CI 1.26 to 1.91) and gestational diabetes (aOR=1.62, 95% CI 1.07 to 2.46), compared with unexposed women (table 3). Low (0.1–0.2 m/s^2^) or medium (0.3–0.4 m/s^2^) exposure was not associated with a significantly increased risk of any outcome. There were no clear associations for women working part time, although there were few cases in the medium exposed (n=18, 22 and 4 cases in the adjusted analyses for preeclampsia, gestational hypertension and gestational diabetes, respectively) and moderately high exposed groups (n=3, 7 and 3 cases, respectively: online supplementary table S2).

**Table 3:** Multivariable-adjusted logistic regression for the association between maternal occupational exposure to whole-body vibrations and pregnancy complications among full-time working women who gave birth to singleton children in Sweden between 1994 and 2014 (n=710 391)

Whole-body vibration exposure (m/s^2^)	Full-time workers			
		Crude OR (95% CI)		Adjusted* OR (95% CI)
Preeclampsia	n (%) cases	n=710 391	n (%) cases	n=646 490
0	23 435 (3.3)	1.00	21 324 (3.4)	1.00
0.1–0.2	394 (3.4)	1.01 (0.91 to 1.12)	348 (3.3)	0.95 (0.84 to 1.07)
0.3–0.4	93 (4.0)	1.20 (0.98 to 1.48)	77 (4.3)	1.20 (0.95 to 1.52)
≥0.5	100 (5.7)	1.75 (1.34 to 2.14)	97 (5.9)	1.76 (1.41 to 2.20)
Gestational hypertension†		n=706 448		n=642 999
0	30 139 (4.4)	1.00	27 506 (4.4)	1.00
0.1–0.2	501 (4.3)	1.00 (0.91 to 1.09)	450 (4.3)	0.94 (0.85 to 1.04)
0.3–0.4	110 (4.8)	1.11 (0.91 to 1.34)	91 (5.1)	1.08 (0.87 to 1.34)
≥0.5	115 (6.7)	1.55 (1.28 to 1.88)	111 (6.8)	1.55 (1.26 to 1.91)
Gestational diabetes		n=707 418		n=643 726
0	6061 (0.9)	1.00	5417 (0.9)	1.00
0.1–0.2	96 (0.8)	0.95 (0.77 to 1.16)	90 (0.9)	0.92 (0.73 to 1.15)
0.3–0.4	18 (0.8)	0.89 (0.56 to 1.42)	14 (0.8)	0.66 (0.39 to 1.14)
≥0.5	28 (1.6)	1.86 (1.28 to 2.70)	26 (1.6)	1.62 (1.07 to 2.46)

*Adjustments: maternal body mass index (<18.5, 18.5–24.9, 25–29.9, ≥30 kg/m^2^), age (years), parity (birth order of the child), smoking habits (non-smoking, 1–9, ≥10 cigarettes/day), education (4 groups based on highest degree: ≤elementary, high-school, higher education <3 years, higher education ≥3 years), nationality (Swedish, European, Other, Unknown/missing), calendar year, exposure to noise (5 categories:<70, 70–74, 75–79, 80–84, ≥85 dB), combustion particles (yes/no), job strain (four groups: low demands and high control, high demands and low control, low demands and high control, and high demands and high control) and physically strenuous work (average of indices ranging from 1 to 6).

†Includes both gestational hypertension and preeclampsia diagnosis. Excluding women with hypertension before pregnancy (International Classification of Diseases (ICD)-9 codes 642 and 642A-D and ICD-10 codes O13, O13.9, O16 and O16.9).

‡Excluding women with type 1 or type 2 diabetes before pregnancy (ICD-9 250, E10–E14, 648A and O24.0–24.3).

**Figure 1:**
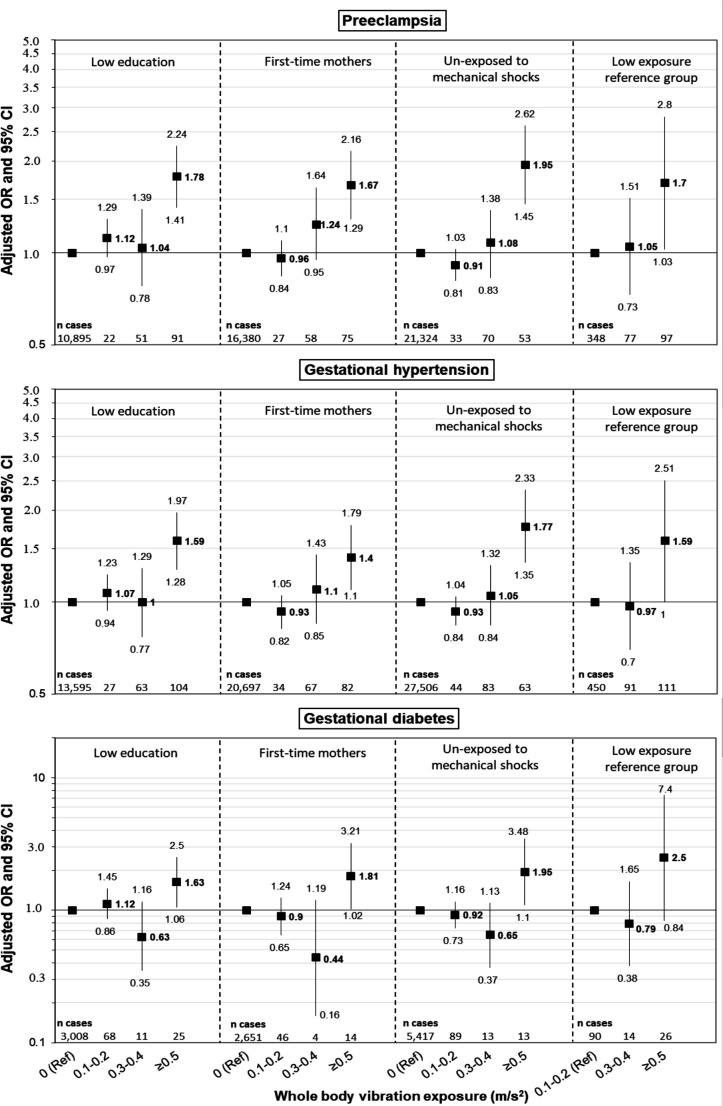
Sensitivity analyses for the associations between maternal occupational exposure to whole-body vibrations and pregnancy complications, restricted to full-time working women who also: (1) had low education (≤high-school), or (2) were first-time mothers, or (3) were unexposed to mechanical shocks, or (4) using the low exposed group as the reference. Adjustments: maternal body mass index, age, parity (for analyses not restricted to first-time mothers) smoking habits, education (only for analyses not restricted to low education), nationality, calendar year, exposure to noise, combustion particles, job strain and physically strenuous work.

In the sensitivity analyses, we first restricted the sample to women without higher education. The associations appeared similar to those for the whole sample of full-time working women, with increased risks in the moderately high exposure group (≥0.5 m/s^2^) compared with the unexposed (preeclampsia: aOR=1.78, 95% CI 1.41 to 2.24; gestational hypertension: aOR=1.59, 95% CI 1.28 to 1.97; gestational diabetes: aOR=1.63, 95% CI 1.06 to 2.50). In further sensitivity analyses, we restricted the sample to full-time workers who were also first-time mothers (figure 1). The associations with exposure to WBV were similar. In addition, we restricted the sample with full-time workers to those who were not exposed to mechanical shocks, which revealed even stronger associations for the moderately high exposure group (≥0.5 m/s^2^) for all outcomes (figure 1). Finally, we excluded the unexposed women from the analyses and thereby replaced the reference group (full-time workers) with the low exposed group (0.1–0.2 m/s^2^). Again, results were similar both for preeclampsia (aOR: 1.70, 95% CI 1.03 to 2.80), gestational hypertension (aOR: 1.59, 95% CI 1.0 to 2.51) and gestational diabetes (aOR: 2.51, 95% CI 0.84 to 7.40; figure 1).

## Discussion

In this nationwide, prospective, cohort study of Swedish women who gave birth between 1994 and 2014, we found that moderately high exposure to WBV was associated with an increased risk of preeclampsia, gestational hypertension and gestational diabetes among full-time workers (n=646 490). The associations were present also when restricting the samples to women with low education, first-time mothers and to those without coexposure to mechanical shocks. Alarmingly, the risks were increased well below the current exposure limit value of 1.15 m/s^2^, at levels around the current action limit value of 0.5 m/s^2^.

Adverse effects of WBV exposure on the female reproductive system have previously been suggested in a review^29^; however, few of the included papers are available in peer-reviewed journals in English. In addition, the mentioned review is focused on birth outcomes like prematurity, spontaneous abortions as well as stillbirth and does not include pregnancy complications. To our knowledge, there is only one previous study that included WBV exposure and pregnancy complications (case control, n cases=102 for preeclampsia and 99 for gestational hypertension), which showed inconclusive results (aOR: 1.2, 95% CI 0.6 to 2.5).^9^ The OR for preeclampsia in the present study was much higher (1.78), possibly due to the more detailed exposure assessment (four exposure groups instead of only yes/no). In addition, we had information about working full or part time, which decreases the risk of underestimating the association due to dilution of the associations by the part-time workers. Indeed, we found no significantly increased risk among part-time workers, while the association was still strong for the full-time working women in all the sensitivity analyses. This also indicates that the increased risk in the full-time working group is related to the WBV exposure rather than some residual confounding factor common to the group of exposed occupations. If such a factor, like poor diet or alcohol consumption, was more common among exposed women, the associations would have been apparent also in the part-time working group as they would be related to the occupational group rather than the WBV exposure. We also found somewhat stronger associations when excluding women who were coexposed to mechanical shocks (to investigate WBV exposure alone). Possibly, these women are more prone to apply for reassignment, resulting in overestimation of their exposure to WBV since we could not take this into account.

The mechanism for an effect of WBV-exposure on pregnancy complications is unknown. Part of the reason for the lacking information in this area is that WBV research has mainly been focused on musculoskeletal disorders, and most studies have been performed on men only.^30^ The increased risk of both the hypertensive disorders and gestational diabetes suggest that an effect of WBV is general, as these disorders are quite different from each other. An experimental study on pregnant rats found that WBV exposure resulted in increased levels of plasma corticosterone and a decreased uterine flow, indicating a stress response after exposure to WBV.^21^ Corticosterone is a major glucocorticoid in rats, while in humans, the main glucocorticoid is cortisol. During pregnancy, the foetus is protected from high circulating cortisol though inhibitory processes in the placenta, where cortisol is converted to its inactive metabolite cortisone. However, in preeclampsia, this conversion is reduced and the level of cortisol is increased to a level higher than the normal pregnancy-related increase.^31^ The function of this conversion is not only to protect the fetus from high levels of circulating cortisol, but also to reduce the vasoconstrictive effects of cortisol in the uterus to allow for the necessary increase in uterine blood flow.^32^ Indeed, a decreased uterine blood flow was identified as one of the characteristics of preeclampsia long ago.^33^

In addition to effects on the uterine blood flow, cortisol has also been shown to affect the maternal glucose metabolism in pregnant sheep, with higher cortisol levels resulting in maternal hyperglycaemia.^34^ Higher cortisol levels (and insulin resistance) have also been reported among pregnant women with gestational diabetes.^35^ Thus, changes in cortisol levels and uterine blood flow could be a possible mechanism for the increased risk of pregnancy complications among the exposed women in the present study. However, this hypothesis is only based on one experimental study with very intense exposure (10 m/s^2^ for 90 min), why endocrine and uterine functions in relation to WBV exposure need to be further studied.

Strengths of the present study include the very large sample size and complete follow-up, the quantitative exposure assessment, as well as the possibility to adjust for many confounders (including many other occupational exposures). Exposure was also assessed prior to the outcome, as the included women provided information regarding occupation already in week 10. Also, the associations appeared strong and robust, and animal studies indicate plausible mechanisms. Together, these strengths are suggestive of causality, but still, we cannot completely exclude residual confounding from, for example, diet and physical activity. A previous study found that sitting a large part of the day may increase the risk of preeclampsia,^36^ which is common among the exposed groups in the present study (mainly drivers). However, the results were consistent even after changing the reference group to the low exposed group (also including a lot of ‘sitting’ occupations), suggesting the associations are not due to sitting per se. Limitations also include the lack of diagnosis date, which meant that we could not control for amount of days away from work (sick leave, parental leave or pregnancy allowance), since we could not know if the absence was due to the outcome or if the outcome was not yet present at the time of the leave. However, including women who were highly absent from work would only result in underestimation of the effect estimates. Finally, there may be some exposure misclassification as we had no information regarding reassignment or changes in tasks that may occur at some point in the pregnancy to reduce certain types of exposure. For flight crew, the exposure to cosmic radiation is required to be reduced by law, which by extension also reduces the exposure to WBV. Thus, excluding this group reduces the risk of exposure misclassification, but for other occupational groups there is no information regarding such changes. Therefore, some of the exposed women were likely less exposed than the levels found in the JEM. This is also a general limitation of the JEM, as there may be some individual variation in exposure within each occupation. The JEM assigns the same value to all women within that occupation, probably resulting in non-differential misclassification. Again, an overestimation or a random misclassification of the exposure would only result in attenuation of the associations, rather than the opposite.

In conclusion, the present study indicates an increased risk of preeclampsia, gestational hypertension and gestational diabetes among women exposed to WBV at full time, even at levels under the current exposure limit value. This indicates that reassignment or pregnancy allowance is a necessary preventive measure for pregnant employees exposed to moderately high levels (≥0.5 m/s^2^) of WBV, although this should be confirmed also by others.
