# Supplementary material for: Occupational exposure to whole-body vibrations and pregnancy complications: a nationwide cohort study in Sweden
Source: Occup Environ Med. 2020 Jun 3;77(10):691–8. doi: 10.1136/oemed-2020-106519 (PMC7509390; doi:10.1136/oemed-2020-106519)
Supplement: Supplementary data [file oemed-2020-106519supp001.pdf]

Supplemental material

Occupational exposure to whole body vibrations and pregnancy complications – a nationwide cohort study in Sweden

Helena Skröder[a]*, Hans Pettersson[b], Maria Albin[a], Per Gustavsson[a], Lars Rylander[c], Filip Norlén[a], Jenny Selander[a]

[a] Unit of Occupational Medicine, Institute of Environmental Medicine, Karolinska Institutet, Box 210, SE-171 77, Stockholm, Sweden.

[b] Section of Sustainable Health, Department of Public Health and Clinical Medicine, Umeå University, SE-901 87, Umeå, Sweden.

[c] Division of Occupational and Environmental Medicine, Institute of Laboratory Medicine, Lund University, Box 188, SE-221 85, Lund, Sweden.

Supplemental Table S1. Characteristics (mean ± SD or %) of mothers who gave birth to singletons in Sweden during 1994-2014 and who worked full time, by exposure to whole body vibrations and mechanical shocks (n=710 391 births).

	**Whole body vibration exposure (m/s^2)**				**Exposure to mechanical shocks**	
	0	0.1-0.2	0.3-0.4	≥0.5	No	Yes
Maternal characteristics	n=694 772 (97.8 %)	n=11 573 (1.6 %)	n=2305 (0.3 %)	n=1741 (0.3 %)	n=708 814 (99.8%)	n=1577 (0.2%)
Age (years) *0% missing*	30.5 ± 4.6	30.5 ± 4.6	30.5 ± 4.5	28.2 ± 4.7	30.5 ± 4.6	29.8 ± 4.6
BMI (%) *7% missing*						
<18.5 kg/m²	2.0	1.2	1.6	2.0	2.0	1.5
18.5-24.9 kg/m²	65.3	62.8	55.0	52.4	65.2	57.1
25-29.9 kg/m²	23.6	26.9	27.8	27.3	23.7	28.1
≥ 30 kg/m²	9.1	9.1	15.6	18.3	9.1	13.3
Parity (birth order of child) *0% missing*	1.6 ± 0.84	1.6 ± 0.83	1.7 ± 0.91	1.6 ±0.88	1.6 ± 0.84	1.6± 0.83
Educational level (%) *<1% missing*						
≤ Elementary	5.9	6.3	7.3	13.6	5.9	10.6
High school	42.8	47.2	60.1	79.6	43.0	55.5
Higher education < 3 years	14.1	32.9	21.2	4.7	14.4	18.6
Higher education ≥ 3 years	37.2	13.6	11.4	2.1	36.7	15.3
Smoking (%) *1% missing*						
Non-smoking	92.8	91.4	92.1	82.3	92.8	87.5
1-9 cigarettes/day	5.3	5.7	4.8	10.7	5.3	7.7
≥10 cigarettes/day	1.9	2.9	3.1	7.0	1.9	4.8
Nationality (%) *0% missing*						
Swedish	94.1	96.7	97.0	96.7	94.2	96.6
European	3.7	2.3	2.4	2.3	3.7	2.4
Other	1.3	0.4	0.0	0.2	1.3	0.1
Unknown	0.9	0.6	0.6	0.8	0.8	0.9
Noise (%) *<1% missing*						
<70 dB	65.0	2.3	0.0	0.0	63.8	0.0
70-74 dB	16.1	94.8	41.0	0.0	17.4	9.4
75-79 dB	5.7	2.1	57.7	88.2	5.8	72.8
80-84 dB	12.0	0.8	0.0	11.8	11.8	17.8
≥85 dB	1.2	0.0	1.3	0.0	1.2	0.0
Mechanical shocks (% Yes/No) *0% missing*	0/100	1.6/98.4	21.7/78.3	51.0/49.0	0/100	100/0
Combustion particles[a] (% Yes/No) *<1% missing*	4.1/95.9	47.0/53.0	65.5/34.5	99.1/0.9	5.1/94.9	78.9/21.1
Job strain (%) *0% missing*						
Active (high demands+high control)	15.3	0.1	21.0	0.0	15.0	29.2
Low strain (low demands+high control)	43.5	42.6	0.0	0.0	43.3	8.7
Passive (low demands+low control)	14.0	12.3	48.3	49.0	14.2	3.0
High strain (high demands+low control)	27.2	45.0	30.7	51.0	27.5	59.1
Physically strenuous work[b] (index 1-6) *0% missing*	2.0 ± 0.7	2.4 ± 0.8	2.6 ± 0.7	3.0 ± 0.3	2.0 ± 0.70	2.7 ± 0.7

[a] Based on exposure to asphalt, diesel, polycyclic aromatic hydrocarbons, lead, and/or other combustion particles.
[b] Average of indices (physically strenuous work) or combination of low/high demands/control (job strain), based on questions about control, demands, turning, lifting, bending, heavy breathing, working with hands above the head or with heavy objects, and repeated movements.

BMJ Publishing Group Limited (BMJ) disclaims all liability and responsibility arising from any reliance placed on this supplemental material which has been supplied by the author(s)

Supplemental Table S2. Multivariable-adjusted logistic regression for the association between maternal occupational exposure to whole body vibrations and pregnancy complications among part-time working women who gave birth to singleton children in Sweden between 1994 and 2014 (n=380 653).

Whole body vibration exposure (m/s^2)	**Part-time workers**			
	n (%) cases	Crude OR (95% CI)	n (%) cases	Adjusted[a] OR (95% CI)
**Preeclampsia**		n=380 653		n=347 810
0	9686 (2.6)	1.00	8809 (2.6)	1.00
0.1-0.2	110 (3.0)	1.17 (0.97, 1.42)	100 (3.0)	1.19 (0.91, 1.38)
0.3-0.4	22 (3.3)	1.28 (0.84, 1.96)	18 (3.2)	1.14 (0.71, 1.85)
≥0.5	4 (1.5)	0.57 (0.21, 1.52)	3 (1.2)	0.41 (0.13, 1.30)
**Gestational hypertension**[b]		n=378 715		n=346 067
0	12 516 (3.3)	1.00	11 428 (3.3)	1.00
0.1-0.2	129 (3.5)	1.06 (0.89, 1.26)	120 (3.6)	1.05 (0.86, 1.27)
0.3-0.4	26 (3.9)	1.18 (0.80, 1.75)	22 (3.9)	1.05 (0.68, 1.63)
≥0.5	8 (3.0)	0.88 (0.44, 1.78)	7 (2.8)	0.72 (0.33, 1.55)
**Gestational diabetes**[c]		n=378 966		n=346 245
0	3721 (1.0)	1.00	3370 (1.0)	1.00
0.1-0.2	37 (1.0)	1.02 (0.74, 1.41)	36 (1.1)	0.99 (0.70, 1.39)
0.3-0.4	5 (0.7)	0.75 (0.31, 1.81)	4 (0.7)	0.58 (0.22, 1.59)
≥0.5	3 (1.1)	1.12 (0.36, 3.48)	3 (1.2)	1.04 (0.32, 3.33)

[a] Adjustments: maternal BMI (<18.5, 18.5-24.9, 25-29.9, ≥30 kg/m^2), age (years), parity (birth order of the child), smoking habits (non-smoking, 1-9, ≥10 cigarettes/day), education (4 groups based on highest degree: ≤ elementary, high-school, higher education<3 years, higher education ≥3 years), nationality (Swedish, European, Other, Unknown/missing), calendar year, exposure to noise (5 categories: <70, 70-74, 75-79, 80-84, ≥85 dB), combustion particles (yes/no), job strain (four groups: low demands and high control, high demands and low control, low demands and high control, and high demands and high control) and physically strenuous work (average of indices ranging from 1-6).

[b] Includes both gestational hypertension and preeclampsia diagnosis. Excluding women with hypertension before pregnancy (ICD-9 codes 642 and 642A-D and ICD-10 codes O13, O13.9, O16, and O16.9).

[c] Excluding women with type 1 or type 2 diabetes before pregnancy (ICD-9 250, E10-E14, 648A, and O24.0-24.3).
